# Protective Effects of Flavonoids Against Mitochondriopathies and Associated Pathologies: Focus on the Predictive Approach and Personalized Prevention

**DOI:** 10.3390/ijms22168649

**Published:** 2021-08-11

**Authors:** Lenka Koklesova, Alena Liskova, Marek Samec, Kevin Zhai, Raghad Khalid AL-Ishaq, Ondrej Bugos, Miroslava Šudomová, Kamil Biringer, Martin Pec, Marian Adamkov, Sherif T. S. Hassan, Luciano Saso, Frank A. Giordano, Dietrich Büsselberg, Peter Kubatka, Olga Golubnitschaja

**Affiliations:** 1 Clinic of Obstetrics and Gynecology, Jessenius Faculty of Medicine, Comenius University in Bratislava, 036 01 Martin, Slovakia; koklesova5@uniba.sk (L.K.); liskova80@uniba.sk (A.L.); marek.samec@uniba.sk (M.S.); kamil.biringer@uniba.sk (K.B.); 2 Department of Physiology and Biophysics, Weill Cornell Medicine in Qatar, Education City, Qatar Foundation, Doha 24144, Qatar; kez4003@qatar-med.cornell.edu (K.Z.); rkmalishaq@hotmail.com (R.K.A.-I.); 3 Lambda Life JSC, 851 01 Bratislava, Slovakia; bugos.ondrej@lambda.sk; 4 Museum of Literature in Moravia, Klášter 1, 664 61 Rajhrad, Czech Republic; sudomova@post.cz; 5 Department of Medical Biology, Jessenius Faculty of Medicine, Comenius University in Bratislava, 036 01 Martin, Slovakia; martin.pec@uniba.sk; 6 Department of Histology and Embryology, Jessenius Faculty of Medicine, Comenius University in Bratislava, 036 01 Martin, Slovakia; marian.adamkov@uniba.sk; 7 Department of Applied Ecology, Faculty of Environmental Sciences, Czech University of Life Sciences Prague, Kamýcká 129, 165 00 Prague, Czech Republic; sherif.hassan@seznam.cz; 8 Department of Physiology and Pharmacology “Vittorio Erspamer”, Faculty of Pharmacy and Medicine, Sapienza University, 00185 Rome, Italy; luciano.saso@uniroma1.it; 9 Department of Radiation Oncology, University Hospital Bonn, Rheinische Friedrich-Wilhelms-Universität Bonn, 53127 Bonn, Germany; frank.giordano@ukbonn.de; 10 European Association for Predictive, Preventive and Personalised Medicine, EPMA, 1150 Brussels, Belgium; 11 Predictive, Preventive, Personalised (3P) Medicine, Department of Radiation Oncology, University Hospital Bonn, Rheinische Friedrich-Wilhelms-Universität Bonn, 53127 Bonn, Germany

**Keywords:** natural substances, phytochemicals, flavonoids, anti-oxidant activity, genoprotection, stress, mitochondrial impairment, mitochondriopathy, mitochondrial function, dysfunction, injury, tumorigenesis, cancer, cardiovascular disease, neurodegeneration, predictive preventive personalized medicine (PPPM/3PM), patient stratification

## Abstract

Multi-factorial mitochondrial damage exhibits a “vicious circle” that leads to a progression of mitochondrial dysfunction and multi-organ adverse effects. Mitochondrial impairments (mitochondriopathies) are associated with severe pathologies including but not restricted to cancers, cardiovascular diseases, and neurodegeneration. However, the type and level of cascading pathologies are highly individual. Consequently, patient stratification, risk assessment, and mitigating measures are instrumental for cost-effective individualized protection. Therefore, the paradigm shift from reactive to predictive, preventive, and personalized medicine (3PM) is unavoidable in advanced healthcare. Flavonoids demonstrate evident antioxidant and scavenging activity are of great therapeutic utility against mitochondrial damage and cascading pathologies. In the context of 3PM, this review focuses on preclinical and clinical research data evaluating the efficacy of flavonoids as a potent protector against mitochondriopathies and associated pathologies.

## 1. Introduction

The terms mitochondrial function and dysfunction are widely employed in bioenergetics and cell biology. Abnormalities in mitochondrial processes, including adenosine triphosphate (ATP) generation, apoptosis, cytoplasmic and mitochondrial matrix calcium regulation, reactive oxygen species (ROS) generation and detoxification, metabolite synthesis, and the intracellular transport, can be termed as mitochondrial dysfunction [1]. Mitochondrial dysfunction affects various organs and tissues, including the brain, muscle, retina, cochlea, liver, and kidney, which are most susceptible to oxidative phosphorylation (OXPHOS) defects. Patients with mitochondrial disorders (mitochondriopathies) exhibit various symptoms, including deafness, visual impairment, heart, liver, and kidney problems, stroke, migraines, diabetes, epilepsy, ataxia, delayed motor and mental development, and failure to thrive, all of which are frequently observed in several non-mitochondrial disorders [2]. Therefore, the effective management of mitochondriopathies is a major challenge in medicine.

Currently, mitochondrial disorders are diagnosed based on functional studies, clinical, biochemical, and histopathologic examinations, and molecular genetic testing [3]. However, diagnostic techniques utilizing cell-free nucleic acids or biofluids such as blood, urine, saliva, cerebrospinal fluid, sweat, or tears could replace invasive tissue biopsies [4,5,6,7]. The paradigm shift from reactive to predictive, preventive, and personalized medicine (3PM) is based on healthcare approaches leveraging targeted preventive measures that account for chronic diseases and ethical as well as economic aspects of medical services [8,9]. 3PM also involves individualized patient profiling, which is important for patient stratification, characterization of individual predisposition, and personalized treatments [10]. Moreover, multi-level diagnostic approaches include molecular biological characterization, novel eHealth-based diagnostic tools, questionnaires, and medical imaging [9]. 

In recent years, the beneficial health effects of flavonoids, naturally occurring polyphenolic compounds, have attracted medical research, including their utilization in pathologies associated with mitochondrial impairments [11] such as cancers, cardiovascular and neurodegenerative diseases [12]. The efficacy of flavonoids is supported by extensive preclinical evidence that represents the basis for further research into the potential future use of these compounds in specific targeted and personalized therapy of mitochondriopathies according to the 3PM approach [13,14,15,16,17,18,19]. 

This review discusses the efficacy of flavonoids in mitochondriopathies such as cancer, cardiovascular diseases (CVDs), and neurodegenerative disorders, highlighting the need for advanced implementation of 3PM.

## 2. Mitochondrial Damage and Associated Impairments

In eukaryotic organisms, mitochondria have an essential role in cellular functions such as energy metabolism, biosynthesis, ionic regulation, oxidation and/or reduction, and signaling pathways associated with cell communication, aging, immune responses, apoptosis, survival, and death [12]. The principal functions of mitochondria are ATP synthesis through OXPHOS, metabolite oxidation by the Krebs cycle, and fatty acid β-oxidation [20]. The mitochondrial genome encodes key electron transport chain (ETC) proteins that play an essential role in energy production in aerobic organisms [21]. Human mitochondrial DNA (mtDNA) is a double-stranded circular molecule consisting of 16,569 base pairs [22]. Under normal conditions, mitochondria contain multiple copies (100 to 10,000 per cell) of their DNA [23].

**Figure 1:**
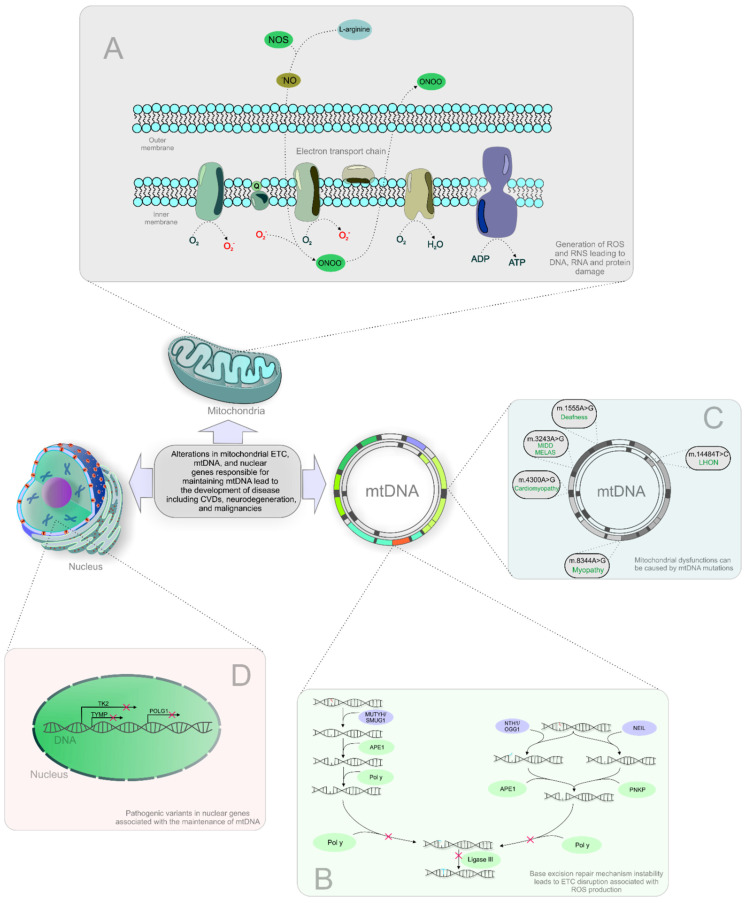
Processes involved in mitochondrial impairments and associated diseases. Abbreviations: ROS, reactive oxygen species; RNS, reactive nitrogen species; mtDNA, mitochondrial DNA; MELAS, mitochondrial encephalomyopathy, lactic acidosis, and strokelike episode syndrome; CPEO, chronic progressive external ophthalmoplegia; CVDs, cardiovascular diseases; NOS, nitric oxide synthase; NO, nitric oxide; ONOO, peroxynitrite; ADP, adenosine diphosphate; ATP, adenosine triphosphate; MUTYH, mutY DNA glycosylase; SMUG1, single-strand selective monofunctional uracil-DNA glycosylase; APE1, human apurinic/apyrimidinic endonuclease; POL *γ*, DNA polymerase subunit gamma; NTH1, Nth like DNA glycosylase 1; OGG1, 8-oxoguanine DNA glycosylase; NEIL, human endonuclease VIII-like; PNKP, polynucleotide kinase 3′-phosphatase; O_2_, dioxygen; O_2_^-^, ion superoxide; H_2_O, dihydrogen monoxide/water.

The ETC is also a source of ROS and reactive nitrogen species (RNS), byproducts of OXPHOS that cause DNA, RNA, and protein damage [24]. The inability of base excision repair (BER) to repair the damaged mtDNA leads to ETC disruption associated with ROS production (shown in Figure 1). Further, the activity of ETC can also act as a predictor and target of drug (venetoclax) sensitivity in multiple myeloma patients [25]. Moreover, oxidative stress and insufficient DNA damage repair could increase DNA damage resulting in mitochondrial dysfunction in patients with depression. Therefore, a marker 8-oxoguanine of oxidative DNA damage obtained from fluid biopsies (blood, urine) could be beneficial for the prevention and prediction of neurodegenerative disorders as mitochondriopathies [26]. Subsequently, extensive oxidative mtDNA damage manifests in several mitochondrial dysfunctions and diseases [27]. Mitochondrial dysfunctions can also be caused by mtDNA mutations, deletions, and impaired DNA replication (shown in Figure 1) [28]. For example, the mtDNA m.3243A>G mutation can lead to clinical phenotypes related to two clinical syndromes: maternally inherited diabetes and deafness (MIDD), and mitochondrial encephalomyopathy, lactic acidosis, and strokelike episode (MELAS) syndrome [29]. Moreover, some clinical features of mitochondrial syndromes associated with mtDNA mutations include a maternal family history due to the maternal pattern of mitochondrial inheritance. The simplicity of analysis of mitochondrial genome sequencing due to the availability of consensus human sequence could help to recognize mtDNA disorders in terms of heredity. Other mtDNA mutations associated with mitochondrial dysfunction are acquired during life by the aging process. These acquired mtDNA mutations are often connected to age-related diseases such as diabetes. Therefore, the progress in the understanding of basic mitochondrial genetics is considered an important tool for analysis of the relationship between inherited mitochondrial mutations and disease phenotypes through the identification of acquired mtDNA mutations [30,31]. Moreover, mitochondrial dysfunction can be caused by pathogenic variants in nuclear genes associated with mtDNA maintenance, including those encoding mtDNA replication enzymes, proteins that function in the maintenance of the mitochondrial nucleotide pool, and proteins that participate in mitochondrial fusion (shown in Figure 1) [32]. Besides, the aging process is connected to a decrease in mitochondrial biogenesis (fusion and fission) and also in a critical process eliminating dysfunctional mitochondria characterized as mitophagy [20]. Moreover, the incidence and frequency of mtDNA mutations increase markedly with age, contributing to cellular senescence [33].

As discussed above, mitochondrial impairments are associated with various highly heterogeneous diseases in terms of their different clinical features and genetic etiology. Therefore, the analysis and/or elucidation of molecular mechanisms associated with mitochondrial impairments can represent the challenges for diagnosis and further clinical management [34]. Finally, mitochondrial dysfunction is a hallmark of many diseases known as mitochondriopathies, including malignancies, CVDs, and neurodegeneration. Therefore, it is imperative to find novel therapies targeting mitochondrial disease mechanisms. 

### 2.1. Mitochondiopaties Are Involved in Cancer Development

Mitochondria have essential functions in apoptotic pathways and the mechanisms of the Warburg phenotype, processes that are closely related to cancer. Mitochondria play an essential role in the intrinsic pathway of apoptotic cell death associated with mitochondrial outer membrane permeabilization, cytochrome *c* release, apoptosome formation, caspase activation, and cell death [35]. Apoptosis evasion is a hallmark of human cancer development. Cancer cells leverage several survival strategies, including the activation of anti-apoptotic and pro-survival signaling through the inhibition of mitochondrial apoptosis. Therefore, intrinsic (mitochondrial) apoptotic pathways represent a promising target of anticancer strategies [36]. 

In 1956, Otto Warburg described the process by which cancer cells sustain rapid proliferation; this process, known as the Warburg effect, is characterized by increased glucose uptake and lactate secretion (aerobic glycolysis) even under normoxic conditions, suggesting that defects in mitochondrial respiration can promote tumorigenesis [37,38]. In mammals, modulation of protein kinases (PK), such as PKL, PKR, PKM1, and PKM2, augments the Warburg effect in cancer cells [39]. Moreover, mtDNA depletion leads to alterations in mitochondrial function in breast, renal, prostate, and other cancers, and age-related diseases, underscores the role of mitochondria in tumorigenesis [40,41,42,43]. Furthermore, various mutations in Krebs cycle enzymes, including succinate dehydrogenase (SDH), fumarate hydratase (FH), and isocitrate dehydrogenase 1 (IDH1) and 2 (IDH2), are described in cancer cells [44]. SDH mutations are associated with hypoxia pathway activation, which can alter mitochondrial fusion and fission, mitophagy, and OXPHOS. In addition, FH and IDH mutations lead to tumor initiation through the repression of cellular differentiation, and IDH1 and IDH2 mutations cause an energy shift in cancer cells [45,46]. Abnormalities in the mentioned Krebs cycle enzymes promote carcinogenesis through the production of onco-metabolites, including 2-hydroxyglutarate and citrate, increased fatty acid β-oxidation, and epithelial-mesenchymal transition (EMT) induction [47].

Moreover, mutations in mtDNA, especially in genes for complexes I, III, IV, and V, which are closely associated with OXPHOS and redox regulation, were observed in endometrial, cervical, breast, and epithelial ovarian cancer cells [44,46,48]. Specifically, mutations in complex I are associated with a higher α-ketoglutarate/succinate ratio, which promotes tumorigenesis through hypoxia-inducible factor 1α (HIF1α) destabilization [49]. Although mutations in mtDNA lead to mitochondrial dysfunction and the potential for cancer development, these mutations also affect nuclear gene expression through retrograde signaling [44]. 

### 2.2. Mitochondrial Dysfunction in Cardiovascular Diseases

CVDs are the leading cause of global mortality and morbidity [50]. Mitochondria have a pivotal role in the homeostasis of the heart. Mitochondrial morphology is responsive to changes in cardiomyocytes [51]. Mitochondrial diseases that preferentially affect the heart are associated with mitochondrial dysfunctions, such as disruptions in OXPHOS or the ETC [52]. Structural and functional alterations in mitochondrial organelles cause ischemic cardiomyopathy, heart failure, and stroke [53].

Furthermore, disruptions in mitochondrial dynamics, including mitochondrial fusion, fission, biogenesis, and mitophagy, lead to the development and progression of CVDs such as diabetic cardiomyopathy, atherosclerosis, damage from ischemia-reperfusion, cardiac hypertrophy, and decompensated heart failure [54]. Several nuclear genes regulating mtDNA maintenance and replication, including mitochondrial transcription factor A (TFAM), mtDNA polymerase γ (*POLG*), and *PEO1* (Twinkle), are altered in CVDs [55]. Besides, mtDNA mutations that dysregulate mtDNA gene expression promote the pathogenesis of stroke and myocardial infarction [56]. Moreover, hypoxia causes changes in cellular mechanisms that lead to oxidative stress and subsequent mitochondrial dysfunction [57].

In patients with atherosclerosis and associated CVDs dysfunctional mitochondria affect cellular respiration and energy production and also act as dangerous ROS generators leading to induction of apoptosis [58]. The accumulation of ROS and RNS in the heart by dysfunctional mitochondria is associated with several CVDs, including cardiomyopathies and heart failure [59,60]. Interestingly, ROS production caused by TFAM dysfunction is related to mtDNA damage and consequent cardiomyocyte cell cycle arrest resulting in lethal cardiomyopathy [61]. Moreover, the prognosis of cardiomyopathy is poor in children with mitochondrial diseases, especially those with mtDNA defects, including the m.3243A>G mutation in mitochondrially encoded tRNA-Leu (UUA/G) 1 (*MT-TL1*), the m.13513G>A mutation in mitochondrially encoded NADH:Ubiquinone oxidoreductase core subunit 5 (*MT-ND5*), the m.8528T>C mutation in the overlapping region of mitochondrially encoded ATP synthase membrane subunits 6 (*MT-ATP*6) and 8 (*MT-ATP8*), the m.3302A>G mutation in *MT-ND1*, the m.1644G>A mutation in mitochondrially encoded tRNA valine (*MT-TV*), and pathogenic mutations in BolA family member 3 (*BOLA3*) and tafazzin *TAZ.* Children with mentioned mitochondrial mutations have a higher risk of cardiomyopathy and associated mortality. Therefore, the genetic analysis with detailed phenotyping of mitochondrial impairments could be useful for the prognosis of cardiomyopathy [62]. Moreover, several nuclear gene mutations can directly affect the mitochondrial respiratory chain and its components. The alterations in genes of complex I (*NDUFS1*, *NDUFS2*, *NDUFS3*), complex IV (*SURF1*, *SCO1*, *SCO2*, *COX10*, *COX15*), complex V (*ATP12*, *TMEM70*), mitochondrial translation (*TACO1*, *EFG1*), and cardiolipin biosynthesis (*TAZ1*) are associated with cardiomyopathy [59]. Furthermore, intermyofibrillar mitochondria represent a well-organized network of long and dense organelles and contractile myofilaments. In heart failure, a disturbance in the physical and chemical interactions between intermyofibrillar mitochondria and sarcoplasmic reticula reduces cardiomyocyte contractility and induces cell death [63]. Moreover, heart failure can be characterized by mitochondrial calcium overload, higher ROS release, and reduced ATP production [64]. During heart failure, calcium overload commonly increases mitochondrial fission and dysfunction. Subsequently, these processes lead to a decrease in the activity of the heart that is characterized by a reduced ability to fill the left ventricle and eject blood to match the body’s demands. This metabolic demand of the heart could be associated with alterations in heart rate, myocardial inotropic state, and myocardial wall tension that in conclusion promote heart injury. The calcium accumulation is also associated with a reduction in mitochondrial energetics (ATP production) that leads to negative changes in ETC and OXPHOS associated with the generation of cell-damaging ROS and apoptosis induction [65]. Furthermore, cardiolipin is a key mitochondrial phospholipid in the inner mitochondrial membrane required for the activity of the ETC. The loss of cardiolipin causes ROS production associated with the disruption of cardiolipin peroxidation and cytochrome *c* release leading to cardiomyocyte apoptosis. In heart failure, this vicious cycle leads to mitochondrial dysfunction and subsequent cardiomyocyte death [66].

### 2.3. Mitochondriopathies in the Neurodegeneration

Normal mitochondrial dynamics are important for maintaining polarity in highly polarized neurons [67,68]. Neuronal cell death in brain disorders (neurodegeneration) and injury (neurotoxicity and ischemia) is connected to various changes in mitochondrial homeostasis and/or function that include traffic, quality control, turnover, bioenergetics, electron transport, and signaling [69]. Neurons depend more on OXPHOS to fulfill their energy demands than other cell types [70]. Neurodegenerative disorders are also characterized by the gradual accumulation of mtDNA mutations that can potentially decrease ETC and ATP production efficiency and increase ROS production [71]. A higher ROS level could cause further mtDNA mutations in a “vicious circle” that leads to cell death [72]. Moreover, abnormalities of the microtubule-associated protein tau (Tau) were observed in various neurodegenerative disorders, including Alzheimer’s disease (AD), Parkinson’s disease (PD), and Pick’s disease [73]. Mitochondrial dysfunction is closely associated with tau pathology in AD; overexpression of hyperphosphorylated and aggregated tau is suggested to damage axonal transport and cause the abnormal distribution of mitochondria [74].

Mitochondrial dysfunction and oxidative stress contribute to AD and PD, the two most common age-related neurodegenerative diseases [71]. AD, a form of senile dementia, is characterized by the accumulation of damaged mitochondria during aging. Extracellular deposition of amyloid β-peptide (Aβ) plaques and the intracellular formation of neurofibrillary tangles (NFTs) occur in the cerebral cortex of AD patients [75]. In AD, oligomers of Aβ with hyperphosphorylated pTau cause the loss of synaptic function and cognitive impairment [76,77]. Several mutations are closely associated with mitochondrial function, including those in the genes encoding β-amyloid precursor protein (APP), presenilin 1 (*PSEN1)* and 2 (*PSEN2),* and apolipoprotein E (APOE4), lead to AD development. Various missense or deletion mutations of mitochondrial APP cause the inherited form of AD [73]. In addition to APP mutations, mutations in *PSEN1* and *PSEN2* are observed in early-onset familial AD [78]. Moreover, the contribution of APOE4 to AD pathogenesis is related to APOE4-mediated alterations of Aβ aggregation and clearance. APOE4 mutations constitute one of the major genetic risk factors for late-onset sporadic AD [79].

Furthermore, the pathological hallmarks of PD include the loss of dopaminergic neurons in the substantia nigra and the presence of misfolded α-synuclein (α-syn) in intra-cytoplasmic inclusions known as Lewy bodies [80]. PD arises from various mitochondrial dysfunctions, including bioenergetic and transcriptional defects, and alterations in dynamics (fusion or fission), size, morphology, trafficking, transport, and movement. Undoubtedly, mutations in mtDNA, nuclear DNA, and mitochondrial proteins are well described in PD [81]. Therefore, mutations or disturbances in E3 ubiquitin ligase (*Parkin)*, α-syn, a parkin-associated protein involved with oxidative stress (*DJ1),* ubiquitin carboxy-terminal hydrolase L1 (*UCHL1*), auxilin (*DNAJC6*), putative serine-threonine kinase (*PINK1*), synaptojanin 1 (*SYNJ1*), serine peptidase 2 (*HTRA2)*, and endophilin A1 (*SH3GL2*) disrupt several mitochondrial functions and may cause PD development [12].

## 3. Flavonoids Classification and Functions

**Figure 2:**
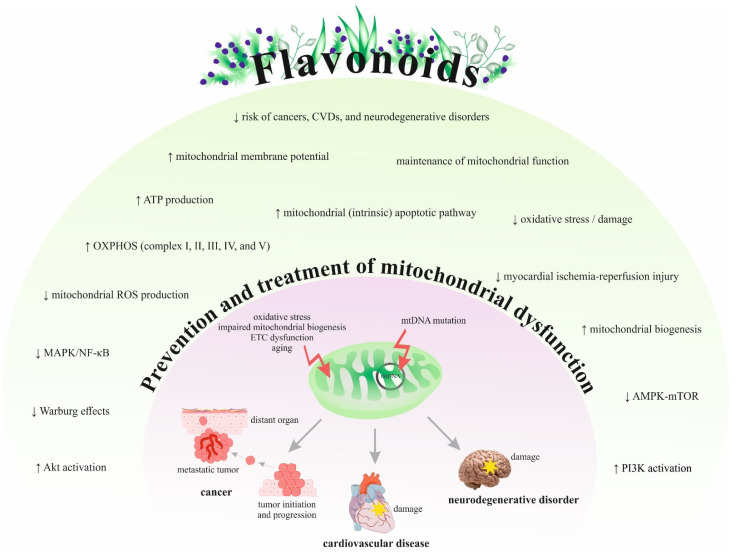
Chemical structures and key representatives of the six major flavonoid classes.

Flavonoids represent an important class of natural substances. All flavonoids are synthesized in plants as bioactive secondary metabolites and contain a basic flavan skeleton that consists of a 15-carbon phenylpropanoid chain (C6-C3-C6 system) with a characteristic polyphenolic structure consisting of two phenyl rings and a heterocyclic pyran ring [82,83]. Flavonoids can be divided into six major groups: isoflavonoids, flavanones, flavanols, flavonols, flavones, and anthocyanidins [84]. Additional minor classes of flavonoids include chalcones, dihydrochalcones, and aurones are categorized into minor flavonoids [85,86]. Moreover, flavonoids are abundant in plant-based foods and are thus consumed through fruits, vegetables, nuts, seeds, grains, bark, roots, stems, flowers, tea, and wine [84]. The general chemical structures [83] and key representatives of the six major flavonoid classes [87,88] are presented in Figure 2. 

Flavonoids have many beneficial properties, such as antioxidant, free radical scavenging, hepatoprotective, cardioprotective, anti-inflammatory, immunomodulatory, antiangiogenic, antiviral, anticancer activities, and antidepressant-like effects [82,89,90,91]. Various flavonoids (vitexin, and baicalin) and other phytochemical compounds such ascurcumin (diarylheptanoid), lycopene (carotene), and ginsenoside (triterpenes), have neuroprotective effects against ischemic-induced injury [92]. Moreover, flavonoids can modulate several key mitochondrial enzymatic pathways [93]. Redox potentials associated with flavonoids’ chemical structure allow these compounds to thermodynamically scavenge ROS, including hydroxyl, superoxide, alkoxyl, alkyl-peroxyl, and nitric oxide radicals [94]. On the other hand, the oxidized reactive byproducts of the redox and scavenging mechanisms of flavonoids chemically destabilize these compounds [95]. Notably, the redox properties of flavonoids vary with the cellular conditions, dosage, treatment time, experimental model, tumorigenic state, and other factors. Under specific cellular conditions such as the occurrence of environmental factors or stressors, the antioxidants can act also as prooxidants. The proxidant activity of flavonoids, e.g., luteolin and fisetin, can be characterized by the ability to undergo autoxidation catalyzed by the transition metals to produce superoxide anions [96,97]. For the determination of prooxidant status is important to evaluate various reductant-oxidant markers such as glutathione (GSH) to GSSG, NADPH to NAPD−, and NADH to NAD− [98]. Prooxidant properties of flavonoids can cause oxidative damage through reactions with different biomolecules, such as lipids, proteins, and DNA [99,100].

Flavonoids generally exhibit low oral bioavailability due to their poor aqueous solubility. The composition of their sources can also affect their bioavailability. Therefore, the gut microbiome is crucial for the absorption and metabolism of flavonoids [101]. Anthocyanidins and pro-anthocyanidins have the lowest bioavailability, while quercetin glucosides, catechin, flavanones, isoflavones, and gallic acid have the highest one [102]. This is the issue that has to be considered from a biotechnological point of view for increasing their bioavailability and facilitating clinical implementation.

Flavonoids provide a valuable contribution in the framework of 3PM. The role of 3PM is to introduce predictive analytical approaches by cost-effective targeted prevention and personalization of medical services. Predispositions and early diagnostics, targeting high-risk individuals, individualized patient profiling, and patient stratification could significantly improve the therapeutic strategies of various diseases [12]. Despite the above-mentioned limitations, flavonoids represent environmentally friendly and cost-effective substances with minimal side effects during long-term administration. Health beneficial effects of flavonoids are promising for 3PM concepts including predictive approaches, targeted prevention, and personalization of medical services, that can positively influence preventive and therapeutic strategies, e.g., anti-cancer effects of flavonoids that can inhibit the initiation of metastasis and their spread in high-risk individuals [86].

## 4. Protective Effects of Flavonoids against Pathologies Associated with Mitochondriopathies

Regular consumption of flavonoids exerts beneficial health effects that can potentially be utilized against several mitochondriopathies, including cancers, CVDs such as atherosclerosis, and neurodegenerative disorders such as AD [103,104].

### 4.1. Preclinical Research

Various in vitro and in vivo studies evaluated the efficacy of flavonoids in mitochondria-associated impairments and/or diseases.

#### 4.1.1. Cancer

Preclinical cancer research demonstrated the potent capacity of flavonoids to modulate pro-carcinogenic mitochondrial dysfunction, especially in signaling cascades associated with the Warburg phenotype and the intrinsic apoptotic pathway. Apigenin (4′,5,7-trihydroxyflavone) blocked cellular glycolysis by inhibiting tumor-specific PKM2 activity and expression in HCT116, HT29, and DLD1 colon cancer cells. Moreover, apigenin treatment decreased the PKM2/PKM1 ratio by blocking the β-catenin/c-Myc/PTBP1 signaling pathway [105]. Furthermore, quercetin suppresses glycolysis by downregulating PKM2, glucose transporter 1 (GLUT1), and lactate dehydrogenase A (LDHA) in MCF-7 and MDA-MB-231 human breast cancer cell lines. Additionally, quercetin treatment inhibited glycolysis and induced autophagy by inhibiting p-Akt/Akt in murine MCF-7 xenografts [106]. Moreover, shikonin treatment inhibited glucose uptake, lactate production, and ATP production in Lewis lung carcinoma and B16 melanoma cells by decreasing PKM2 activity and consequently reversing the Warburg effect [107]. Furthermore, the enzyme hexokinase 2 (HK2) converts glucose to glucose-6-phosphate in the first step of glucose metabolism [108] and promotes the Warburg effect in cancer cells [109]. However, xanthohumol downregulated HK2 and glycolysis and subsequently increased cytochrome *c* release to activate the intrinsic (mitochondrial) apoptotic pathway in the HT29, SW480, LOVO, HCT116, and SW620 colorectal cancer cell lines [13]. The apoptosis-inducing factor (AIF), a mitochondrial protein, is implicated in caspase-independent programmed cell death following its translocation to the nucleus [110]. In an in vitro investigation using multiple biochemical assays, xanthohumol was detected to cause proliferation inhibition and death of the rat glioma C6 cells (in a time- and dose-dependent manner) via a mechanism of inducing AIF pathway apoptosis by triggering mitochondrial stress [111]. Impressively, pyruvate dehydrogenase kinase 1 (PDK1) is a gatekeeper of glycolysis and mitochondrial OXPHOS; its inhibition can reverse the Warburg phenotype of tumor cells [112]. Licochalcone A suppressed HIF1α, GLUT1, and PDK1 in HCT116 colorectal cancer, H1299 non-small cell lung carcinoma, and H322 primary bronchioalveolar carcinoma cells. Besides, higher intracellular oxygen content resulting from the direct inhibition of mitochondrial respiration was observed after licochalcone A treatment [113]. Furthermore, EGCG promoted mitochondrial depolarization and suppressed glycolysis in 4T1 murine breast cancer cells, as demonstrated through reduced levels of glucose, lactate, ATP, HIF-1α, and GLUT1. EGCG also inhibited several glycolytic enzymes, including HK, phosphofructokinase, LDH, and PK, in the same model [14]. Moreover, albanol B, a benzofuran flavonoid, exerted potent anti-cancer effects by inducing apoptosis through mtROS production and associated increased phosphorylation of Akt and extracellular signal-regulated kinase 1/2 (ERK1/2) in A549, BZR, H1975, and H226 human lung cancer cell lines. The anti-cancer potential of albanol B was associated with the induction of apoptosis and G_2_/M phase cell cycle arrest through mtROS production [114]. Lysionotin, a bioactive flavonoid from *Lysionotus pauciflorus* Maxim., has been shown in a combined in vitro (HepG2 and SMMC-7721 cells) and in vivo (HepG2 and SMMC-7721-xenograft tumor mouse model) experiment the ability to exert remarked anti-liver cancer properties through a mechanism that causes caspase-3 mediated mitochondrial apoptosis pathway. The outcomes of this study have also revealed that lysionotin could control oxidative stress, which was found to be involved in lysionotin-mediated mitochondrial apoptosis by regulating nuclear factor erythroid 2–related factor 2 (Nrf2) signaling pathway [115]. BAS-4, a prenylated flavonoid (isolated from the Amazon plant *Brosimum acutifolium*), was observed to cause anticancer properties against the C6 glioma cells by promoting apoptosis mediated by mitochondrial transmembrane potential loss and Akt pathway disruption [116]. Furthermore, treatment with isoquercitrin (25 µM), a bioactive flavonol, exhibited anti-cancer effects against SK-Mel-2 human melanoma cells, and the mechanism was observed to be related to its effect on mitochondria-mediated apoptosis. Various mechanisms were reported, including the reduction in the levels of procaspase-8 and -9, and Bcl-2 protein, and the enhancement of cleaved PARP and Bax expressions. The caspase-independent mitochondrial-mediated apoptosis was found to be linked to the increase of AIF and Endo G protein expressions. Besides, the anti-proliferative activity was determined to be associated with the downregulation of the PI3K/Akt/mTOR signaling pathway [117]. In a mechanistic study using in vitro (A549 cells) and in silico assays, the flavonoid myricetin (73 µg/mL) showed the capacity to induce anticancer properties against lung cancer cells by promoting cell cycle arrest and ROS-reliant mitochondria-facilitated apoptosis [118]. Moreover, the flavonoid silibinin, a bioactive substance from *Silybum marianum*, exerted a cytotoxic effect against SCC-25 human oral squamous carcinoma cells. The in vitro assay disclosed the mechanism of action via inducing apoptosis by releasing mitochondrial cytochrome *c* into the cytosol following by activating caspases-3 and -9 [119].

As demonstrated in the above-discussed preclinical studies, flavonoids have the potential to reverse the Warburg effect by targeting signaling molecules associated with mitochondrial respiratory defects. Moreover, the anti-Warburg effect of flavonoids could be multiplied by an antioxidant, anti-inflammatory, ROS scavenging, immunomodulatory, anti-angiogenic [82], and other anti-cancer activities such the participation in cell cycle arrest, apoptosis induction, autophagy, and suppression of cancer cell proliferation and invasiveness [83].

#### 4.1.2. Cardiovascular Diseases

Flavonoids potently affect the complex pathways associated with CVD-related mitochondrial dysfunctions. Nuclear factor-κB (NF-κB), a transcription factor, regulates many cellular processes, including immunity, inflammation, and cell survival. Besides, NF-κB signaling is also essential for mitochondrial processes, such as biogenesis, metabolism, and apoptosis [120]. Further, NF-κB is a redox-sensitive transcription factor because ROS can regulate its activity. An extract of *Aronia melanocarpa* rich in polyphenols, especially anthocyanins, activated NF-κB by ROS production in human aortic endothelial cells (HAECs), resulting in potential cardioprotection [121]. Moreover, the peroxisome proliferation-activated receptor (PPAR) family regulates mitochondrial function, turnover, and energy metabolism. Therefore, PPAR activity can represent a therapeutic target to restore impaired mitochondrial function [122]. Cornelian cherry (*Cornus mas* L.) fruits rich in anthocyanins, phenolic acid, flavonols, and iridoids decreased serum triglyceride levels and increased PPARα protein expression in the liver, suggesting protective effects on diet-induced hypertriglyceridemia and atherosclerosis in a hypercholesterolemic rabbit model. Moreover, increased expression of PPAR in the liver indicated its hypolipidemic effect obtained from enhanced fatty acid catabolism which subsequently led to decreased triglyceride levels [123].

Interestingly, mitochondrial dysfunction contributes to myocardial ischemia-reperfusion-induced cardiomyocyte apoptosis. Yu et al. recently reported that naringenin can alleviate myocardial ischemia-reperfusion injury by reducing mitochondrial oxidative stress damage, cytochrome *c* release, and oxidative markers. Moreover, mitochondrial biogenesis was maintained by increased nuclear respiratory factor 1 (NRF1), TFAM, and OXPHOS II, III, and IV subunit complexes in vitro (H9c2 cardiomyoblasts) and in vivo (rats) models [15]. Moreover, mitochondrial dysfunction has a crucial role in the pathogenesis of fructose-induced cardiac hypertrophy. The bioflavonoid naringin inhibited mtROS production and thereby relieved mitochondrial dysfunction in H9c2 rat myoblasts after fructose exposure and high fructose-induced cardiac hypertrophy. Indeed, the suppression of cardiomyocyte hypertrophy by naringin was mediated through downregulation of the AMP-activated protein kinase (AMPK)-mechanistic target of rapamycin (mTOR) signaling axis [124].

Furthermore, proteins involved in mitochondrial dynamics, including mitofusin 2 (Mfn2), mitochondrial dynamin-like GTPase (OPA1), dynamin-related protein 1 (Drp1), and fission 1 (Fis-1), regulate mitochondrial homeostasis under stress conditions [125]. Treatment of myocardial ischemic mice with 7,8-dihydroxyflavone (7,8-DHF) reversed cardiac dysfunction and cardiomyocyte abnormalities through the suppression of mitochondrial fission, as demonstrated by decreased protein levels of Fis-1. Besides, 7,8-DHF improved the mitochondrial membrane potential and reduced mitochondrial superoxide levels in hydrogen peroxide (H_2_O_2_)-treated H9c2 rat myoblasts. 7,8-DHF also prevents mitochondrial fission by inhibiting proteolytic cleavage of OPA1 in H9c2 cells [126]. Similarly, 7,8-DHF improved cardiac function and inhibited cardiac injury mediated by increased OPA1 protein expression, Akt activation, OXPHOS, and mitochondrial membrane potential dysregulation in doxorubicin-induced cardiotoxicity in Kunming mice and H9c2 cells [127].

In many cases, diabetic cardiomyopathy causes heart failure. Dihydromyricetin increased mitochondrial function in streptozotocin-induced diabetic mice, as demonstrated by increases in ATP content, citrate synthase activity, and complex Ι, ΙΙ, ΙΙΙ, ΙV, and V activities [128]. Moreover, quercetin protected mitochondria by restoring the cellular redox balance after isoproterenol-induced cardiac hypertrophy in mice. Quercetin attenuated cardiac hypertrophy by increasing sulfhydryl group availability and mitochondrial superoxide dismutase activity and reducing mitochondrial permeability transition pore opening in the same model [129]. Impressively, intraperitoneal injection of luteolin in mice with lipopolysaccharide-induced myocardial injury mitigated mitochondrial injury and oxidative stress by decreasing AMPK phosphorylation in septic heart tissue and stabilizing the mitochondrial membrane potential. In summary, luteolin attenuates lipopolysaccharide-induced myocardial injury associated with mitochondrial impairments in mice through the inhibition of apoptosis and enhancing autophagy via modulation of AMPK signaling [16]. Furthermore, icariin, a prenylated flavonol glycoside, protected H9C2 cardiomyocytes from oxidative stress by scavenging ROS and promoting ERK pathway phosphorylation. Icariin also preserved Ca^2+^ homeostasis and mitochondrial membrane potential stability [130]. Moreover, cyanidin, an anthocyanin pigment, improved mitochondrial function in mice with lipopolysaccharide-induced myocardial injury by reducing oxidative damage through the associated factor Opa1 and the antioxidant gene thioredoxin-1 (Trx1) [131]. Tilianin, a natural flavonoid glycoside, is known for its cardioprotective effect against myocardial ischemia/reperfusion injury (MIRI). In a comprehensive preclinical study, the mechanism of action of this compound has been determined through hindering Ca^2+^/calmodulin-dependent protein kinase II (CaMKII)-mediated mitochondrial apoptosis and c-Jun *N*-terminal kinase (JNK)/NF-κB inflammation [132]. Moreover, the cardioprotective effect of fisetin, a natural flavonoid, has been comprehensively investigated in a combined experiment (*in vitro*, *in vivo*, and *in silico*). The results showed that treatment with fisetin could suppress mitochondrial oxidative stress and mitochondrial dysfunction and repress glycogen synthase kinase 3β (GSK3β) activity, where the induced effects were reported as possible mechanisms of action [133]. In another animal study, the administration of fisetin (20 mg/kg) attenuated the myocardial infarct size, apoptosis, lactate dehydrogenase, and creatine kinase in serum/perfusate of the rat hearts subjected to ischemia/reperfusion injury. The results concluded that phosphoinositide 3-kinase (PI3K) activation is needed to mediate fisetin-associated cardioprotection against ischemia/reperfusion injury in rat heart [134]. Furthermore, phosphorylation of Drp1 at serine 616 is associated with increased Drp1 enzyme activities that consequently contribute to cell death. It is known that myocardial injury after cardiac arrest (CA) leads to critical myocardial dysfunction and death, including mitochondrial dysfunction. In this regard, baicalin, a natural flavonoid molecule, was studied in vivo for its cardioprotection against CA-induced injury by regulating mitochondrial dysfunction. Male Sprague-Dawley rats were treated with baicalin (100 mg/kg, administered intragastrically once daily for 4 weeks) and the results proved that this compound has potently reduced mitochondrial dysfunction and exhibited cardioprotective effect after CA by a mechanism via inhibiting the phosphorylation at serine 616 and translocation of Drp1 and excessive fission of mitochondria. In conclusion, the inhibition of Drp1-mediated mitochondrial fission might be the possible mechanism of baicalin in preventing CA-induced myocardial injury [135].

Several preclinical (*in vitro* and *in vivo*) studies indicate that flavonoids can reverse CVD-associated mitochondriopathies by targeting various molecules and signaling pathways.

#### 4.1.3. Neurodegenerative Disorders

Aluminum, a neurotoxicant, causes oxidative damage as observed in various neurodegenerative disorders such as AD [136]. However, naringin reduced the neurotoxic effects of aluminum in rats. The administration of a higher dose of naringin (80 mg/kg) significantly improved cognitive performance, reduced mitochondrial oxidative damage, and downregulated certain mitochondrial enzymes, including NADH dehydrogenase, succinate dehydrogenase, and cytochrome oxidase, compared to control aluminum-treated rats [137]. APP and Aβ co-localize in mitochondria; Aβ inhibits the respiratory chain, and altered mitochondrial function can result in changes in APP and eventual alterations in the production of amyloidogenic derivatives [138]. Nevertheless, quercetin reduced Aβ and BACE1-mediated cleavage of APP in a murine triple transgenic AD model (3xTg-AD) [139]. Treatment with quercetin also decreased ROS levels and restored normal mitochondrial morphology in hippocampal neurons affected by H_2_O_2_-induced neuronal toxicity and Aβ-induced neurodegeneration this suggests that quercetin could prevent neuronal mitochondrial dysfunction [140].

Furthermore, quercetin upregulated protein kinase D1 (PKD1), Akt, cAMP response-element binding protein (CREB), and the CREB target gene *BDNF—*all of which are associated with mitochondrial dysfunction related to neurodegenerative disorders [141,142]—in murine MN9D dopaminergic cells. Besides, quercetin increased the mitochondrial bioenergetic capacity and protected MN9D cells against 6-hydroxydopamine (6-OHDA)-induced neurotoxicity [143]. Interestingly, acetylcholinesterase activity causes mitochondrial impairments; however, cholinesterase inhibitors increase mitochondrial biogenesis through AMP-Activated PK in the hippocampus [144]. Mitochondrial γ-secretase participates in the metabolism of mitochondria-associated APP [145]. In this regard, a meta-analysis of 17 preclinical studies on AD animal models revealed that EGCG exerts neuroprotective effects by reducing acetylcholinesterase activity, enhancing α-, β-, and γ-secretase activity, decreasing Aβ_42_ levels and tau phosphorylation, and modulating anti-oxidative, anti-inflammatory, and anti-apoptotic processes [146]. Moreover, the flavonoid isoquercitrin enhanced mitochondrial function by attenuating mitochondrial membrane potential loss, downregulating the outer mitochondrial membrane voltage-dependent anion channel (VDAC), and preventing mtROS accumulation in a model of streptozotocin-induced AD in murine Neuro-2a neuroblastoma cells [18]. Two other flavonoids, mangiferin, and morin, alleviated A*β*-induced mitochondrial impairments such as decreased respiratory capacity, mitochondrial membrane depolarization, and cytochrome *c* release in cortical neurons in the AD model [147].

Quercetin increased mitochondrial complex I activity (demonstrated by increased NADH oxidation), constraining mtROS production in a rotenone-induced rat model of PD [17]. Recently, the neuroprotective effect of quercetin has been investigated in 6-OHDA-treated PC12 rat pheochromocytoma cells and the 6-hydroxydopamine (6-OHDA)-lesioned rat model of PD. The outcomes of in vitro assay showed that treatment with quercetin (20 μM) promoted mitochondrial quality control, diminished oxidative stress, boosted the levels of the mitophagy markers (*Parkin* and *PINK1*), and lowered α-syn protein expression in 6-OHDA-treated PC12 cells. Moreover, the results of in vivo test proved that treatment of PD rats with quercetin (10 mg/kg/day and 30 mg/kg/day) for two weeks by oral gavage has led to producing progressive PD-like motor behaviors, alleviate neuronal death, and lessen mitochondrial damage and α-syn accumulation. All experimental results assumed that the neuroprotective effect of quercetin was defeated by the knockdown of both *PINK1* and *Parkin* [148]. Furthermore, in PC12 rat adrenal gland cells, the naturally occurring hydroxy flavonoid myricitrin ameliorated 6-OHDA–induced mitochondrial damage through the inhibition of mitochondrial oxidation, as demonstrated by reduced ROS production and lipid peroxidation in rat brain mitochondria [149]. Myricitrin also mitigated mitochondrial dysfunction by increasing DJ-1 activity in SN4741 substantia nigra dopaminergic cells with 1-methyl-4-phenylpyridinium-induced mitochondrial dysfunction [150]. Another study revealed that hesperidin, a citrus flavanol, exerted antioxidative and antiapoptotic properties by maintaining mitochondrial function against rotenone-induced apoptosis in an SK-N-SH neuroblastoma cellular model of PD [151]. 

The mechanism of the neuroprotective effect of tilianin against cerebral ischemia using oxygen-glucose deprivation (OGD) protocol was detailed, where tilianin was found to affect mitochondrial function and inflammation by alleviating CaMKII-dependent mitochondrion-mediated apoptosis and MAPK/NF-κB inflammatory activation following cellular OGD injury [152]. In traditional Chinese medicine, hydroxysafflor yellow A (HSYA; a C-glucosyl quinochalcone that belongs to the flavonoid family) has been widely employed as a protective agent against ischemia/reperfusion injury. This compound has also been noticed to reduce the levels of ROS and suppress cellular apoptosis. In a mechanistic study, HSYA was found to decrease phenylalanine levels and promote mitochondrial function via the upregulation of mitochondrial fission protein Drp1, leading to causing a neuroprotective effect against cerebral ischemia/reperfusion injury [153]. A recent in vivo study using male Sprague Dawley rats was designed to assess the protective effects of HSYA-mediated mitochondrial permeability transition pore (mPTP) on cerebral ischemia/reperfusion injury and its mechanism. The obtained results indicated that HSYA treatment remarkably enhanced brain microvascular endothelial cells (BMECs) viability, lowered the production of ROS, the opening of mPTP, and translocation of cytochrome *c*. HSYA was also detected to potentiate MEK and boost phosphorylation of ERK expression in BMECs, hinder apoptosis mediated by mitochondrial, and repress cyclophilin D (CypD). Interestingly, HSYA has been found to decrease the infarct size in animal models [154]. Nobiletin, a polymethoxylated flavonoid, is commonly detected in the genus *Citrus*. In a multiple biochemical investigation, nobiletin was found to regulate mitochondrial dysfunction mediated by the ETC system downregulation by hindering complex I and III in pure mitochondria and the cortical neurons of rats. This molecule at various concentrations in micromolar ranges was noticed to potently reduce mitochondrial ROS production, restrain apoptotic signaling, improve ATP production, and improve neuronal viability under conditions of complex I repression. The induced effect was related to the downregulation of translocation of AIF, and the upregulation of complex I activity, and the expression of antioxidant factors such as Nrf2 and heme oxygenase 1 (HO-1). Based on the acquired data, this study suggested that nobiletin might have promising neuroprotective action against neurodegenerative diseases such as AD and PD [155].

As discussed above, flavonoids can alleviate mitochondrial impairments mainly by reducing ROS or maintaining mitochondrial functions; these abilities can improve cognitive function associated with the two most common neurodegenerative disorders, AD and PD (Table 1).

### 4.2. Clinical Data

In addition to preclinical studies, clinical research also highlights the efficacy of flavonoids in the etiopathology of mitochondriopathies, including cancers, CVDs, and neurodegenerative disorders.

#### 4.2.1. Cancer

Despite the beneficial effects of flavonoids elucidated in preclinical cancer studies, no clinical studies to date have directly focused on the mechanistic effects of flavonoids on mitochondrial impairments. Otto Warburg hypothesized that mitochondrial dysfunction initiates cancer formation characterized by decreased glycolytic energy production in contrast with mitochondrial respiration [156]. Targeted therapies using flavonoids against the Warburg effect could have important applications in future cancer management [157]. Flavonoid supplements could support cancer prevention, especially at high-risk individuals; key risk factors include obesity (due to low physical activity and/or sedentary lifestyle) [158,159], stress exposure [160], Flammer syndrome [161], accelerated aging processes [162], and chronic inflammation [163]. Moreover, genetic predispositions [164], the early detection of mitochondrial impairments [156], and the detection of cancer with metastatic potential [165] are highly predictive in cancer management. Therefore, individualized patient profiling is an essential tool for cancer predisposition and early diagnostics [166]. In evaluating the applications of flavonoids in patient stratification and individualized therapy, it is essential to consider the varying mechanisms underlying cancer, as cancers associated with mitochondrial impairments may differ from those associated with nuclear mutations [167,168,169].

Eventually, the application of plant-derived natural substances such as flavonoids alone or in combination with anticancer drugs could constitute a promising strategy against the Warburg phenotype within the 3PM framework.

#### 4.2.2. Cardiovascular Diseases

Mitochondria play a significant role in the pathogenesis of various CVDs. However, current clinical research aimed at finding novel molecules applicable against CVDs focuses primarily on the general protective properties of flavonoids rather than their direct impact on mitochondrial impairments. 

Isoflavone treatment for 12 weeks reduced serum high-sensitivity (hs)-C-reactive protein (CRP) levels and improved brachial flow-mediated dilatation in patients with clinically manifested atherosclerosis and prior ischemic stroke [170]. Moreover, dietary intake of flavonoid-rich foods can prevent mitochondriopathies related to CVDs. Flavonoids, including flavonols, flavones, flavanones, anthocyanidins, and proanthocyanidins, significantly decreased the risk of CVD mortality [171]. Interestingly, flavonoids in black, green, herbal, and berry teas possess protective effects against various CVDs, including stroke, myocardial infarction, and coronary heart diseases [172].

Moreover, transthyretin amyloidosis is a rare progressive systemic disease characterized by increased left ventricular wall thickness and diastolic dysfunction. In many cases, this disease leads to amyloidotic transthyretin mitochondrial cardiomyopathy [173]. After 12 months of treatment with green tea and its extracts, in which EGCG is abundant, echocardiography revealed no changes in cardiac wall thickness and mass progression, suggesting that green tea exerts protective effects against amyloidotic transthyretin mitochondrial cardiomyopathy [174]. Furthermore, menopause in women is often related to the aging process and higher CVD risk with possible mitochondrial connections [175,176]. In women with early menopause, supplementation with soy protein and isoflavones significantly decreased various CVD risk markers [177]. 

Moreover, altered mitochondrial functions also cause hyperinsulinemia, glucose intolerance, dyslipidemia, obesity, and elevated blood pressure, collectively known as metabolic syndrome [178]. Blueberries rich in flavonoids decreased plasma oxidized low-density lipoprotein (LDL), serum malondialdehyde, and hydroxynonenal concentrations in patients with metabolic syndrome. These results suggest that blueberries have cardioprotective effects and alleviate metabolic syndrome [179]. Furthermore, cranberries (*Vaccinium macrocarpon* Ait.) rich in polyphenols, including flavonoids and ellagic acid, increased plasma antioxidant capacity and reduced lipid oxidation by decreasing oxidized LDL and malondialdehyde in women with metabolic syndrome [180].

Furthermore, mitochondrial structure and/or function alterations are associated with a higher risk of various CVDs, including ischemic cardiomyopathy, heart failure, and stroke [53]. Therefore, higher intake of fruit-based flavonoids, especially through anthocyanin-rich (cyanidin, delphinidin, malvidin, pelargonidin, petunidin, peonidin) and flavanone-rich (eriodictyol, hesperetin, naringenin) foods, reduced the risk of nonfatal myocardial infarction and ischemic stroke in men [181]. Flavonoids also have potential in the secondary prevention of ischemic heart disease. Flavonoids in chokeberry (*Aronia melanocarpa*) extract reduced serum 8-isoprostane, oxidized LDL, hsCRP, and monocyte chemoattractant protein-1 (MCP-1) levels and increased adiponectin levels in patients who survived myocardial infarction and had received statin therapy [182]. In conclusion, current clinical studies provide predominantly general data on the efficacy of flavonoids against CVDs rather than precise mechanisms related to mitochondrial function.

#### 4.2.3. Neurodegenerative Disorders

Neurodegenerative disorders are closely associated with mitochondrial deregulation [69]. Flavonoids can potently attenuate the negative impact of mitochondrial dysfunction on the pathogenesis of neurodegenerative disorders, as shown by preclinical research. However, current clinical studies primarily offer results dealing with the general effects of flavonoids on neurodegenerative diseases. 

Increased cellular oxidative stress leads to α-syn accumulation and subsequently to mitochondrial dysfunction [183]. The flavonoid EGCG inhibits α-syn aggregation and reduces associated toxicity. Therefore, EGCG treatment can potentially delay or prevent various mitochondriopathies associated with neurodegenerative disorders [184]. However, EGCG treatment did not modify the progression of multiple system atrophy, a neurodegenerative disease associated with α-syn aggregation in neurons and oligodendrocytes. In addition, higher doses (1200 mg) were connected to hepatotoxic effects in several patients [185].

Moreover, mitochondrial dysfunction is associated with impaired homocysteine metabolism, which leads to aging tissue degeneration [186]. Therefore, elevated plasma homocysteine levels are typical in AD patients in a moderate phase compared to AD patients in the initial and control groups. A polyphenol-rich antioxidant drink decreased plasma total homocysteine levels in AD patients, especially in the moderate phase [187]. The flavonoid-rich *Ginkgo biloba* extract (EGb 761^®^) improved cognition, daily living, and social behavior in patients with uncomplicated AD or multi-infarct dementia—which are both associated with mitochondrial impairments [188]. Furthermore, the administration of EGCG in patients delayed the progression of multiple system atrophy-associated disabilities [189].

Although beneficial effects of flavonoids were observed in the mentioned clinical studies, detailed mechanisms concerning mitochondrial impairments were not evaluated. Therefore, current clinical research indicates significant positive effects of flavonoids on neurodegenerative diseases, but the direct effects of flavonoids on mitochondrial function remain unclarified. Table 2 provides a detailed overview of the discussed clinical studies on the role of flavonoids in the etiopathology of mitochondriopathies, including cancer, CVDs, and neurodegenerative disorders.

Although the beneficial effects of flavonoids reinforce the hypothesis that the consumption of these naturally occurring compounds could be responsible for health benefits, the evidence from the clinical sphere is still limited and there are doubts about their use in practice. Different results of the efficacy of flavonoids in clinical studies could be based on different patient profiling (age, gender, race, or financial status) e.g., in the meta-analysis of Peters et al. (2001) [172] or a small number of participants in studies of Kristen et al. (2012) [174]. The use of flavonoids could be also associated with various side effects. A study by Levin et al. (2019) described the hepatotoxic effects after treatment with a higher dose of EGCG (1200 mg) in several patients [185]. Therefore, the determination of accurate doses of flavonoids for patients could mitigate the side effects and increase their safety. Moreover, the limitations of several studies are connected to the insufficient investigation of more precise molecular mechanisms of the efficacy of flavonoids. Nevertheless, as demonstrated in our previous studies [12,72] biomarkers obtained from liquid biopsy could facilitate the prediction and diagnosis of mitochondrial impairments. Further, liquid biopsy could replace the invasive tissue biopsies associated with various health risks. Despite some limitations, flavonoids still represent environmentally friendly and cost-effective substances suitable for long-term use, and more precise evaluations of the effects of flavonoids, along with individualized patient profiling and patient stratification, could improve medical strategies for the prevention and treatment of mitochondriopathies.

**Figure 3:**
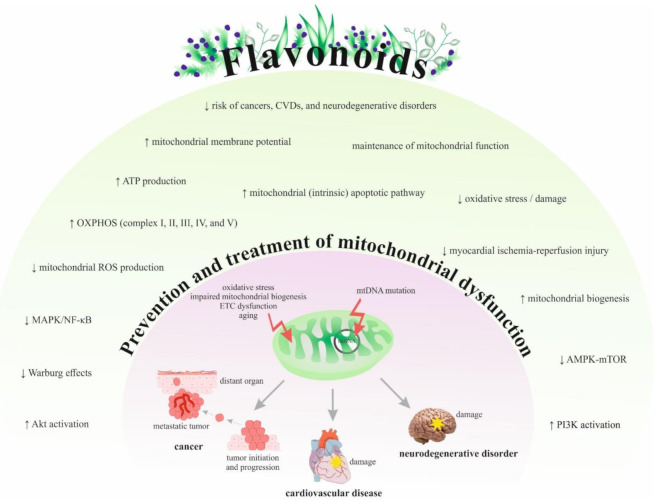
The mechanisms of flavonoids in the prevention and treatment of mitochondriopathies. Abbreviations: EGCG, epigallocatechin-3-gallate; EGb 761^®^, *Ginkgo biloba* extract; CVDs, cardiovascular diseases; mtDNA, mitochondrial DNA; OXPHOS, oxidative phosphorylation; ROS, reactive oxygen species; **↑,** increase/induce; ↓, decrease/reduce; ETC, electron transport chain.

Figure 3 summarizes the efficacy and potential of flavonoids in preventing or treating various mitochondriopathies related to cancer, CVDs, and neurodegenerative disorders based on preclinical and clinical findings.

## 5. Conclusions

Recent progress in 3P medicine demonstrates that patient stratification and individualized patient profiling are instrumental for cost-effective targeted prevention and treatments tailored to the person [4,5,7,9]. Individualized evaluation of the mitochondrial impairments [190,191] is essential for the risk assessment related to mitochondriopathies and associated pathologies, including but not restricted to cancer, CVDs, and neurodegenerative disorders [192,193,194]. Targeting mitochondrial homeostasis is a promising innovation in the overall therapeutic strategy.

The treatment and prevention of diseases in patients with mitochondriopathies have attracted a lot of attention in current research, novel therapeutic strategies. Contextually, flavonoids, naturally occurring polyphenolic compounds are of particular interest exerting significant health benefits in primary, secondary, and tertiary care protecting against stress overload, genotoxicity, mitochondrial dysfunction, and associated pathologies [195,196,197,198,199].

Both preclinical and clinical studies demonstrate flavonoids as highly protective agents reducing mitochondrial impairments and mitigating risks of associated pathologies. To improve individual outcomes and to increase cost-efficacy, the 3PM approach is strongly recommended to implement these benefits in healthcare providing new opportunities for prevention and treatment of stress-related disorders, oncology, cardiology, and neurology, amongst others [4,5,7,9,200,201].

## Figures and Tables

**Table 1:** Flavonoids targeting deregulated mitochondrial processes associated with cancer, CVDs, and neurodegenerative diseases in preclinical research.

Flavonoid	Mitochondrial Disorder	Study Design	Effects	Ref
Cancers				
Apigenin	colon cancer	HCT116, HT29, and DLD1 colon cancer cells	↓ PKM2 activity and expression, ↓ PKM2/PKM1 ratio, blocked β-catenin/c-Myc/PTBP1	[105]
Quercetin	breast cancer	MCF-7 and MDA-MB-231 human breast cancer cell lines; murine MCF-7 xenografts	↓ PKM2, ↓ GLUT1, ↓ LDHA, ↓ p-Akt/Akt	[106]
Shikonin	lung carcinoma and melanoma	Lewis lung carcinoma and B16 melanoma cells	↓ glucose uptake, ↓ lactate production, ↓ tumor cell ATP production, ↓ PKM2	[107]
Xanthohumol	colorectal cancer	HT29, SW480, LOVO, HCT116, and SW620 colorectal cancer cell lines	↓ HK2, ↓ glycolysis, ↑ cytochrome *c,* ↑ intrinsic (mitochondrial) apoptotic pathway	[13]
	Glioma	rat glioma C6 cells	↓ proliferation, ↑ apoptosis, ↑ AIF	[111]
Licochalcone	colorectal cancer, non-small cell lung carcinoma, and primary bronchioalveolar carcinoma	HCT116 colorectal cancer, H1299 non-small *cell* lung carcinoma, and H322 primary bronchioalveolar carcinoma cells.	↓ HIF-1α, ↓ GLUT1, ↓ PDK1, ↑ intracellular oxygen content, ↓ mitochondrial respiration	[113]
EGCG	breast cancer	4T1 mouse breast cancer cells	↓ glucose, ↓ lactate, ↓ ATP levels, ↓ HIF1α, ↓ GLUT1, ↑ mitochondrial depolarization, ↓ HK, ↓ phosphofructokinase, ↓ LDH, ↓ PK	[14]
Albanol B	lung cancer	A549, BZR, H1975, and H226 human lung cancer cell lines	↑ mtROS production, ↑ phosphorylation of Akt, ↑ phosphorylation of ERK1/2, ↑ apoptosis, ↑ cell cycle arrest at G_2_/M phase	[114]
Lysionotin	liver cancer	HepG2 and SMMC-7721 cells and HepG2 and SMMC-7721-xenoghraft tumor mouse model	↑ mitochondrial apoptosis pathway, ↑ caspase 3, control oxidative stress	[115]
BAS-4	Glioma	C6 glioma cells	↑ apoptosis, loss of mitochondrial membrane potential, Akt pathway disruption	[116]
Isoquercetin	Melanoma	SK-Mel-2 human melanoma cells	↑ mitochondrial apoptosis, ↓ procaspase-8 and -9, ↓ Bcl-2 protein, ↑ cleaved caspase, ↑ Bax, ↑ AIF, ↑ Endo G, ↓ PI3K/Akt/mTOR signaling	[117]
Myricetin	lung cancer	*in vitro* (A549 lung cancer cells) and in silico study	↑ cell cycle arrest, ↑ ROS-reliant mitochondrial apoptosis	[118]
Silibinin	oral squamous carcinoma	SCC-25 human oral squamous carcinoma cells	↑ apoptosis, ↑ cytochrome c, ↑ caspase-3 and -9	[119]
**Cardiovascular Diseases**				
Extract of *Aronia melanocarpa*	cardiovascular disease	50 μg/mL of *Aronia Melanocarpa* fruit extract in human aortic endothelial cells	↑ NF-κB, ↑ ROS production	[121]
Cornelian cherry fruits	hypertriglicerydemia and atherosclerosis	diet-induced hypertriglicerydemia and atherosclerosis in a New Zealand rabbit model	↓ serum triglyceride levels, ↑ PPARα protein expression	[123]
Naringenin	myocardial ischemia-reperfusion injury	Sprague-Dawley rats and H9c2 cardiomyoblasts	↓ mitochondrial oxidative stress damage, ↓ mitochondrial cytochrome *c* release, ↓ oxidative markers, ↑ mitochondrial biogenesis, ↑ NRF1, ↑ TFAM, ↑ OXPHOS II, III and IV subunits, ↓ myocardial ischemia-reperfusion injury	[15]
Naringin	cardiomyocyte hypertrophy	H9c2 rat myoblasts after fructose exposure, high fructose-induced cardiac hypertrophy	↓ mtROS production, ↓ cardiomyocyte hypertrophy, ↓ AMPK-mTOR	[124]
7,8-Dihydroxyflavone	myocardial ischemia	adult Kunming mice, H_2_O_2_-treated H9c2 *Rattus norvegicus* myoblasts	↓ mitochondrial fission, ↓ Fis-1, ↑ mitochondrial membrane potential, ↓ mitochondrial superoxide, ↓ OPA1	[126]
	heart disease	doxorubicin—induced cardiotoxicity in Kunming mice; H9c2 cells	↑ cardiac function, ↓ cardiac injury, ↑ OXPHOS, ↑ mitochondrial membrane potential, ↑ Akt activation, ↑ OPA1	[127]
Dihydromyricetin	diabetic cardiomyopathy	streptozotocin—induced diabetic C57BL/6J mice; dihydromyricetin treatment at 100 mg/kg/day	↑ ATP content, ↑ citrate synthase activity, ↑ complex Ι, ΙΙ, ΙΙΙ, ΙV, and V activities	[128]
Quercetin	cardiac hypertrophy	isoproterenol—induced cardiac hypertrophy in male Swiss mice	↑ protein sulfhydryls, ↑ superoxide dismutase activity, ↓ opening of mitochondrial permeability transition pore	[129]
Luteolin	sepsis-induced cardiomyopathy	intraperitoneal injection of luteolin in male C57BL/6 mice; lipopolysaccharide—induced myocardial injury	↓ mitochondrial injury, ↓ oxidative stress, ↓ phosphorylation of AMPK in septic heart tissue, ↓ destabilized mitochondrial membrane potential	[16]
Icariin	cardiac oxidative stress injury	H9C2 cardiac myocytes; H_2_O_2_—induced oxidative stress injury	↑ ROS scavenging, phosphorylation of ERK pathway, maintenance of Ca^2+^ homeostasis, prevention of mitochondrial membrane potential dissipation	[130]
Cyanidin	sepsis and myocardial oxidative or inflammation-induced injury	lipopolysaccharide—induced myocardial injury in male/female C57BL/6 mice	↑ mitochondrial function, ↓ oxidative damage, ↓ Opa1, ↓ Trx1	[131]
Tilialin	myocardial ischemia/reperfusion injury	oxygen-glucose deprivation/reperfusion-injured H9c2 cardiomyocytes; ischemia/reperfusion- (I/R-) injured isolated rat hearts	↑ CaMKII-mediated mitochondrial apoptosis, ↑ JNK/NF-κB inflammation	[132]
Fisetin	acute myocardial infarction	*in vitro* (cardiomyocytes), in vivo (rat heart model), and in silico experiment	↓ mitochondrial oxidative stress, ↓ mitochondrial dysfunction, ↓ GSK3β activity	[133]
	ischemia/reperfusion injury	Male Wistar rat model	↓ myocardial infarct size, apoptosis, lactate dehydrogenase, and creatine kinase in serum/perfusate, ↑ PI3K activation	[134]
Baicalin	myocardial injury after cardiac arrest	Cardiac arrest-induced injury in male Sprague-Dawley rats	↓ mitochondrial dysfunction, ↑ cardioprotective effect, ↓ phosphorylation at serine 616 and translocation of Drp1, and excessive fission of mitochondria	[135]
**Neurodegenerative Disorders**				
Naringin	neurodegenerative disorders and neurotoxicity	Six-week administration of aluminum (100 mg/kg) and naringin (40 and 80 mg/kg) to male Wistar rats	↑ cognitive performance, ↓ mitochondrial oxidative damage, ↓ NADH dehydrogenase, ↓ succinate dehydrogenase, ↓ cytochrome oxidase	[137]
Quercetin	AD	Quercetin injection every 48 h for 3 months (25 mg/kg) in a murine triple transgenic AD model (3xTg-AD)	↓ Aβ, ↓ BACE1-mediated cleavage of APP	[139]
		H_2_O_2_—induced neuronal toxicity and Aβ—induced neurodegeneration of hippocampal neurons	↓ ROS, ↑ recovery of normal mitochondrial morphology	[140]
EGCG		meta-analysis of 17 preclinical studies; animal models	↓ Aβ_42_ level, ↓ acetylcholinesterase activity, ↓ tau phosphorylation, anti-oxidation, anti-inflammation, anti-apoptosis, ↑ α-, β-, and γ-secretase activity	[146]
Isoquercitrin		streptozotocin—induced mitochondrial dysfunction and oxidative stress in murine Neuro-2a neuroblastoma cells	↑ mitochondrial function, ↑ mitochondrial membrane potential, ↓ VDAC, ↓ mtROS	[18]
Mangiferin and morin		A*β—*induced mitochondrial impairments in cortical neurons from E18 Sprague-Dawley rat embryos	↓ mitochondrial impairments, ↑ respiratory capacity, ↓ mitochondrial membrane depolarization, ↓ cytochrome *c* release	[147]
Quercetin	PD	Murine MN9D dopaminergic cells; *6*-*hydroxydopamine*—induced neurotoxicity	↑ phosphorylation of PKD1, Akt, CREB, BDNF; ↑ mitochondrial bioenergetics capacity	[143]
		6-hydroxydopamine—treated PC12 rat pheochromocytoma cells; 6-hydroxydopamine—lesioned rat model of PD	↑ mitochondrial quality control, ↓ oxidative stress, ↑ *Parkin,* ↑ *PINK1*, ↓ α-syn	[148]
		rotenone—induced rat model (inbred adult Sprague–Dawley rats)	↑ mitochondrial complex-I activity, ↓ ROS	[17]
Myricitrin		PC12 rat adrenal gland cells, 6-OHDA—induced mitochondrial damage and neurotoxicity	↓ mitochondrial damage, ↓ mitochondrial oxidation, ↓ ROS, ↓ lipid peroxidation	[149]
		1-methyl-4-phenylpyridinium—induced mitochondrial dysfunction in SN4741 substantia nigra dopaminergic cells	↑ maintenance of mitochondrial function, ↑ DJ-1	[150]
Hesperidin		rotenone—induced apoptosis in SK-N-SH neuroblastoma cells	↑ maintenance of mitochondrial function	[151]
Tilialin	cerebral ischemia	oxygen-glucose deprivation protocol; in silico docking mode and SH-SY5Y human-derived thrice cloned cell line	↑ mitochondrial function, ↓ inflammation, ↓ CaMKII-dependent mitochondrion-mediated apoptosis, ↓ MAPK/NF-κB inflammatory activation	[152]
Hydroxysafflor yellow A	ischemia/reperfusion injury	PC12 rat adrenal gland cells	↓ ROS, ↓ suppress cellular apoptosis, ↓ phenylalanine levels, ↑ mitochondrial function, ↑ upregulation of mitochondrial fission protein Drp1	[153]
	cerebral ischemia/reperfusion injury	male Sprague Dawley rats	↑ brain microvascular endothelial cells viability, ↓ ROS, ↓ opening of mitochondrial permeability transition pore, ↓ translocation of cytochrome *c*, ↑ phosphorylation of ERK, ↓ cyclophilin D	[154]
Nobiletin	mitochondrial dysfunction in AD and PD	mitochondrial dysfunction mediated by the ETC system downregulation by hindering complex I and III in cortical neurons of rats	↓ mtROS, ↓ apoptosis, ↑ ATP production, ↑ neuronal viability, ↓ translocation of AIF, ↑ complex I, ↑ Nrf2, ↑ HO-1	[155]

**Explanatory notes:** ↑ increased/improved, ↓ decreased/inhibited. **Abbreviations:** PKM2, protein kinase M2; c-Myc, MYC proto-oncogene; PTBP1, polypyrimidine tract binding protein 1; GLUT1, glucose transporter 1; LDHA, lactate dehydrogenase A; Akt, serine/threonine-protein kinases; ATP, adenosine triphosphate; HK2, hexokinase 2; HIF-1α, hypoxia-inducible factor 1-alpha; PDK1, phosphoinositide-dependent kinase-1; LDH, lactate dehydrogenase; PK, protein kinase; mtROS, mitochondrial reactive oxygen species; ERK1/2, extracellular signal-regulated kinases 1/2; NF-κB, nuclear factor-κB; PPARα, peroxisome proliferator-activated receptor α; NRF1, nuclear respiratory factor 1; TFAM, transcription factor A mitochondrial; OXPHOS, oxidative phosphorylation; AMPK, 5’ AMP-activated protein kinase; mTOR, mechanistic target of rapamycin; Fis-1, mitochondrial fission 1 protein; OPA1, mitochondrial dynamin like GTPase; Trx1, thioredoxin; NADH, nicotinamide adenine dinucleotide hydrogen; Aβ, amyloid beta; BACE1, beta-site APP cleaving enzyme 1; APP, amyloid-beta precursor protein; CREB, cAMP-response element binding protein; BDNF; brain-derived neurotrophic factor; tau, microtubule associated protein; AD, Alzheimer’s diseasAbbreviationse; PD, Parkinson’s disease, EGCG, epigallocatechin-3-gallate; H_2_O_2_, hydrogen peroxide; AIF, apoptosis-inducing factor; GSK3β, glycogen synthase kinase 3β; HO-1, heme oxygenase 1; CaMKII, Ca^2+^/calmodulin-dependent protein kinase II; Nrf2, nuclear factor erythroid 2–related factor 2; Drp1, dynamin-related protein; JNK, c-Jun *N*-terminal kinase.

**Table 2:** Effects of flavonoids in the etiology of mitochondriopathies (cancer, CVDs, and neurodegenerative disorders).

Flavonoid	Supposed Mitochondriopathy	Clinical Trial	Study Design	Effect	Ref
**Cardiovascular Diseases**					
Isoflavone	ischemic stroke	randomized, double-blinded, placebo-controlled trial	12-week treatment; 80 mg/day of isoflavone (*n* = 50) or placebo (*n* = 52)	↓ serum hsCRP	[170]
Flavonols, flavones, flavanones, anthocyanidins, and proanthocyanidins	CVDs	meta-analysis (22 prospective studies)	100 mg/day of flavonoids	↓ risk of all-cause mortality, ↓ risk of CVD mortality	[171]
Tea consumption	CVDs, stroke, myocardial infarction	meta-analysis (10 cohort studies and 7 case-control studies)	3 cups per day	in 4 studies: ↑ risk of coronary heart disease, ↑ myocardial infarction In the remaining studies: ↓ myocardial infarction, ↓ coronary heart disease	[172]
EGCG	amyloidotic transthyretin cardiomyopathy	randomized placebo-controlled clinical trial	12-month treatment, patients with amyloidotic transthyretin cardiomyopathy (*n* = 19); 547 ± 49 mg/day of EGCG	no changes in cardiac wall thickness and mass	[174]
Soy protein with isoflavones	CVDs	randomized controlled trial	6-month treatment with 15 g of soy protein and 66 mg of isoflavones/day during early menopause in Caucasian women from Hull and East Riding in Yorkshire (*n* = 200)	↓ fasting glucose, ↓ fasting insulin, ↓ insulin resistance, ↓ systolic blood pressure	[177]
Blueberries	CVD risk	randomized controlled trial	8-week treatment with 50 g of freeze-dried blueberries/daily in participants with metabolic syndrome (*n* = 48)	↓ plasma oxidized LDL, ↓ serum malondialdehyde, ↓ hydroxynonenal	[179]
Cranberries	CVD risk	randomized, double-blind, placebo-controlled trial	8-week treatment in women with metabolic syndrome (*n* = 15–16/group); 1st group: cranberry juice (480 mL/day); 2nd group: placebo (480 mL/day)	↑ plasma antioxidant capacity ↓ lipid oxidation, ↓ oxidized LDL, ↓ malondialdehyde	[180]
Fruit-based flavonoids (flavanones and anthocyanins)	CVDs, myocardial infarction, and ischemic stroke	Health Professionals Follow-Up Study	men in analysis (*n* = 43,880)	↓ risk of nonfatal myocardial infarction and ischemic stroke	[181]
Chokeberry flavonoid extract (*Aronia melanocarpa* E) and statin therapy	ischemic heart disease	double-blind, placebo-controlled, parallel trial	6-week combination therapy with statin (simvastatin) and flavonoids from chokeberry extract (3 × 85 mg/day) or placebo in patients (*n* = 44) who survived myocardial infarction	↓ serum 8-isoprostanes, ↓ oxidized LDL, ↓ hsCRP, ↓ MCP-1, ↑ adiponectin	[182]
**Neurodegenerative Disorders**					
EGCG	multiple system atrophy	randomized, double-blind, placebo-controlled clinical trial (NCT02008721)	48-week treatment; 92 participants-EGCG treatment (*n* = 47) and placebo (*n* = 45)	no modification in disease progression	[185]
Antioxidant drink rich in polyphenols (apple and lemon juice concentrate, apple and green tea extracts, and vitamins B and C)	AD	multicenter, randomized, double-blind controlled clinical trial	8-month treatment; 100 participants: AD patients (*n* = 24 in initial phase, *n* = 24 in moderate phase) and healthy controls (*n* = 52)	↓ total plasma homocysteine levels in AD patients	[187]
EGb 761^®^	uncomplicated AD and multi-infarct dementia	double-blind, placebo-controlled trial	26-week treatment with a 120-mg dose (40 mg t.i.d.) of EGb 761; placebo group (*n* = 161) and EGb treated group (*n* = 166)	↑ cognitive assessment, ↑ daily living, ↑ social behavior	[188]
EGCG	multiple system atrophy	prospective, randomized, double-blind, placebo-controlled parallel-group phase III, multicenter study	48-week treatment in patients (*n* = 86); PROMESA protocol	↓ progression of multiple system atrophy-associated disabilities	[189]

**Explanatory notes:** ↑ increased/improved; ↓ decreased/inhibited. **Abbreviations:** CVD, cardiovascular disease; hsCRP, serum high-sensitivity C-reactive protein; EGCG, epigallocatechin-3-gallate; LDL, low-density lipoprotein; MCP-1, monocyte chemoattractant protein-1; AD, Alzheimer’s disease; EGb 761^®^, *Ginkgo biloba* extract.

## Data Availability

Not applicable.
